# Service-Oriented Node Scheduling Scheme for Wireless Sensor Networks Using Markov Random Field Model

**DOI:** 10.3390/s141120940

**Published:** 2014-11-06

**Authors:** Hongju Cheng, Zhihuang Su, Jaime Lloret, Guolong Chen

**Affiliations:** 1 College of Mathematics and Computer Science, Fuzhou University, Fuzhou 350108, China; E-Mails: zhihuangsu@126.com (Z.S.); fzucgl@163.com (G.C.); 2 Department of Computer Science, The University of Alabama, Tuscaloosa, AL 35401, USA; 3 Instituto de Investigación Para la Gestión Integrada de Zonas Costeras, Universidad Politécnica de Valencia, C/ Paranimf n° 1, Grao de Gandia, Gandia, Valencia 46730, Spain; E-Mail: jlloret@dcom.upv.es; 4 Fujian Provincial Key Laboratory of Network Computing and Intelligent Information Processing, Fuzhou University, Fuzhou 350108, China

**Keywords:** wireless sensor networks, service-oriented, multi-service, node scheduling, Markov Random Field, energy efficiency

## Abstract

Future wireless sensor networks are expected to provide various sensing services and energy efficiency is one of the most important criterions. The node scheduling strategy aims to increase network lifetime by selecting a set of sensor nodes to provide the required sensing services in a periodic manner. In this paper, we are concerned with the service-oriented node scheduling problem to provide multiple sensing services while maximizing the network lifetime. We firstly introduce how to model the data correlation for different services by using Markov Random Field (MRF) model. Secondly, we formulate the service-oriented node scheduling issue into three different problems, namely, the multi-service data denoising problem which aims at minimizing the noise level of sensed data, the representative node selection problem concerning with selecting a number of active nodes while determining the services they provide, and the multi-service node scheduling problem which aims at maximizing the network lifetime. Thirdly, we propose a Multi-service Data Denoising (MDD) algorithm, a novel multi-service Representative node Selection and service Determination (RSD) algorithm, and a novel MRF-based Multi-service Node Scheduling (MMNS) scheme to solve the above three problems respectively. Finally, extensive experiments demonstrate that the proposed scheme efficiently extends the network lifetime.

## 1. Introduction

Wireless sensor network is emerging as an important technology which includes a large number of self-organized sensor nodes connected with wireless communications [1]. The sensor nodes are generally equipped with sensing devices, micro-processor, limited memory, wireless transmitter and lower-energy batteries. The characteristics of the sensor networks, such as cheapness and self-organization, make it possible to deploy them in various applications, such as environment data collection, smart home, and machine health monitoring. At the same time, the rapid development of sensor technology leads to the emerging sensor nodes equipped with multiple sensing devices such as temperature, sound and motion, *etc.* One important trend for the future wireless sensor networks is to provide various sensing services. In the future service-oriented wireless sensor networks, one single node can simultaneously support multiple sensing services [2–9] rather than a single sensing service. How to solve the problem of resources sharing while providing different sensing services in an energy-efficient way is one of the most important problems in the future service-oriented wireless sensor network.

The node scheduling scheme is an important way to solve the problem of resource sharing between different sensing services and extend network lifetime [10–16]. In some applications, sensed data should be collected from all nodes in the network, which is not an efficient way to collect all raw sensed data for the application, especially in densely deployed wireless sensor networks. It is generally tolerant if the final collected data for different services is within the given error thresholds since there are deviations between the raw sensed data and the physical values of the monitored targets [9,10]. Furthermore, the sensed data is generally correlated and redundant between nodes that provide one single service or different services since they observe the same targets in the same geographic region [15–20]. Therefore, a subset of representative nodes can be selected and every representative node provides its determined services within error thresholds guarantees, while the rest data is not sent to the application. This strategy can not only reduce the energy cost and prolong the network lifetime, but also contribute to solve some other issues in densely deployed wireless sensor networks [15], such as lower network throughput, transmission conflicts, *etc.* It shall be mentioned that the multi-service node scheduling problem also determines the sensing services that every representative node should provide when they are selected.

**Figure 1.**
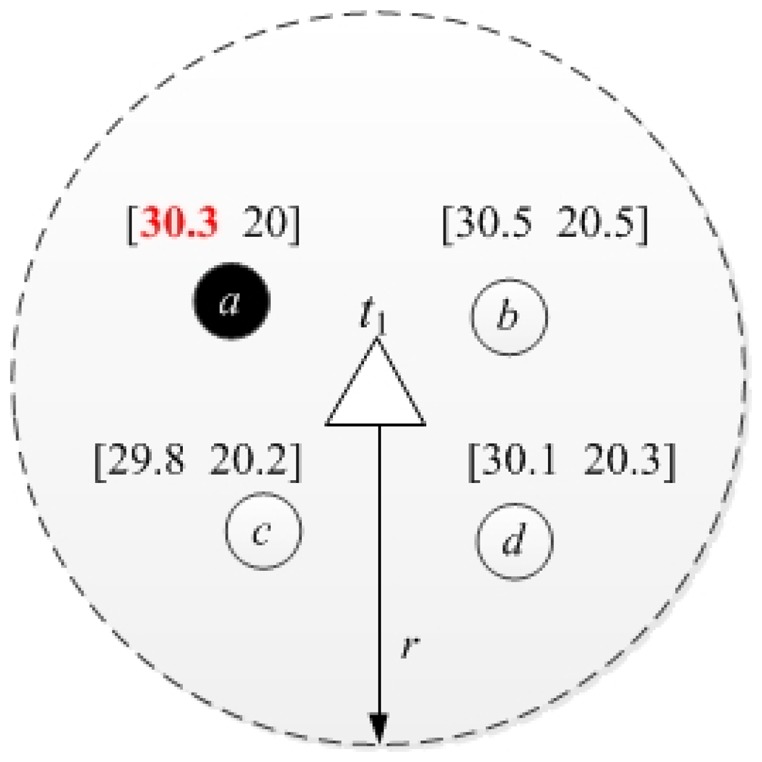
An example of the representative node selection and the provided services in the wireless sensor network.

Figure 1 shows a simple network with one monitored target *t*_1_ whose sensing radius is denoted as *r*. And target *t*_1_ falls in the sensing radius of four nodes, *a*, *b*, *c* and *d*. Two kinds of services, *s*_1_ and *s*_2_, are provided by each node in the network, and the sensed data for services *s*_1_ and *s*_2_ are listed above the node, *i.e.*, the sensed data of node *a* is [30.3 20]. Note that the sensed data is a noisy version of the physical value, and the physical values of services *s*_1_ and *s*_2_ are generally correlated. Assume that the physical value of service *s*_1_ is 10 less than that of *s*_2_ and the error threshold for the two services is 0.5. The selected representative node is marked with a black solid circle and the service data is marked in red color. As we can see, the observed data of node *a*, *b*, *c* and *d* for service *s*_1_ is 30.3, 30.5, 29.8 and 30.1 accordingly. Obviously, the last three values are within the error threshold of the first one. Furthermore, the sensed data of node *a* for service *s*_1_ is 30.3, and we can infer that the sensed data for *s*_2_ is 20.3 since the two services are correlated. Note that the observed data of node *a*, *b*, *c* and *d* for service *s*_2_ is 20, 20.5, 20.2 and 20.3, and these data is within the error threshold of 20.3 too. In this way, node *a* can be selected as the representative node to provide two services and only the data for service *s*_1_ is required since the rest data is within the error threshold.

**Figure 2.**
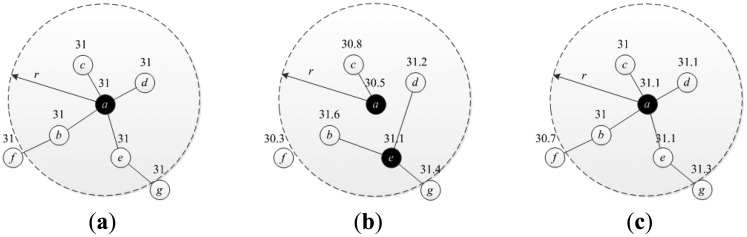
An example of the representative node selection scheme: (**a**) using noise-free data; (**b**) using noise-corrupted data; (**c**) using denoised data.

However, it is not proper to select the representative nodes and determine the services they provided directly by the sensed data because it is a noisy version of the realistic data due to the affection of the environment, node parsimony and other factors [21]. Here we demonstrate an example to illustrate this issue. Assume that every node provides one service data and the error threshold is 0.5. Figure 2a–c show the results for the representative node selection problem with the noise-free data, noise-corrupted data and denoised data separately. There is one link between two nodes if they monitor the same target and the deviation between the data is smaller than the given error threshold. As we can see in Figure 2, the selected representative node set is {*a*}, {*a*, *e*} and {*a*} with noise-free, noise-corrupted and denoised data accordingly. In this example, the number of selected nodes with noise-corrupted data is larger than that with noise-free and that with denoised data, while they are the same with the latter two data. However, it is almost impossible to obtain the noise-free data in practice. In this paper, we introduce a novel idea to obtain the denoised data which is described in Section 4.2.

The efficiency of data denoising depends heavily on how to model the data correlation between the sensed data for one single service or multiple services. However, the sensed data in the wireless sensor networks has the property of Markov dependence due to the spatial continuous variation in the practical environment [22,23]. In this case, Markov Random Field (MRF) model is an important statistical tool to describe the distribution and correlation of data for one service or between two different services in the multi-dimensional domains via the probability functions. In this paper, we adopt the MRF model to describe the correlation of data for one service or two separate services, and propose a novel algorithm to denoise the sensed data for multiple services. The main contributions of this paper are summarized as follows:

— We introduce the Markov Random Field (MRF) model to describe the correlation of data for one service or two separate services in wireless sensor networks;
— We formulate the multi-service data denoising problem which aims at minimizing the noise level of sensed data, the representative node selection problem concerning with selecting a number of active nodes while determining the services they provide, and the multi-service node scheduling problem which aims at maximizing the network lifetime in the service-oriented wireless sensor networks;
— We propose a Multi-service Data Denoising (MDD) algorithm and a novel multi-service Representative node Selection and service Determination (RSD) algorithm to efficiently select the representative node and their provided services;
— We also present a novel MRF-based Multi-service Node Scheduling (MMNS) scheme based on the above two algorithms for the multi-service node scheduling problem to enlarge the network lifetime.

The rest of this paper is organized as follows: in Section 2, we present the related works. Section 3 describes the system model and the problem formulation. In Section 4, we describe the proposed scheme. In Section 5, we analyze the theoretical performance of our proposed algorithms. Section 6 presents simulation results and Section 7 presents the conclusions.

## 2. Related Works

The service-oriented network is one of the most important trends for the future wireless sensor network. It leads to a lot of challenging issues including the system architecture and other optimizing problem with this new architecture [16]. The node scheduling problem provides a new idea to extend the network lifetime by exploiting the characteristics of dense deployment and the correlation of data for multiple services.

Some researchers proposed the general theoretical models for the service-oriented sensor networks. Gracanin *et al.* [2] proposed a service-centric model by viewing the wireless sensor networks as service providers. They also provided a general framework to express/evaluate the capabilities and functionalities of wireless sensor networks. Rezgui and Eltoweissy [3] introduced the service-oriented sensor-actuator networks which provide sensing and actuation services to any application rather than provide sensing and actuation capabilities to one specific application.

Some researchers have focused on building a service-oriented platform/middleware, which plays an important role in facilitating the design, development, and implementation of service-oriented wireless sensor network [4–6]. Mohamed and Al-Jaroodi [4] surveyed the current challenges and requirements of service-oriented middleware in wireless sensor network and reviewed some representative approaches. Edgardo Avilés-López *et al.* [5] proposed a service-oriented architecture called TinySOA, which provides a high-level abstraction for the development of applications in wireless sensor networks. TinySOA allows programmers to access the wireless sensor networks from their applications using a service-oriented API. Zhang *et al.* [6] designed an open community-oriented platform aiming to support federated sensor data as a service, featuring interoperability and reusability of heterogeneous sensor data and data services.

An important design goal in the service-oriented sensor networks is to provide data of various services by satisfying the requirement of applications. Geyik *et al.* [7] proposed a graph-based model for describing sensor services and formulated the process of dynamic sensor service composition as a cost-optimization problem which aims to minimize the total cost of component services. Wang *et al.* [8] studied the cross-layer sleep scheduling problem to prolong the network lifetime while satisfying the service availability requirement on the application layer. They proved that the problem is NP-hard, and proposed two approximation algorithms based on LP relaxation. Cheng *et al.* [9] exploited the spatial correlation between multiple service data and aimed at selecting a minimum number of active nodes to provide services with data accuracy guaranteed [9]. However, the proposed algorithms did not consider the energy efficiency and might lead to bad performance.

The sensed data of neighbors is generally correlated in densely deployed wireless sensor networks. This characteristic is helpful to reduce the transmitted data in the network and improve the energy efficiency [19,20,23]. Vuran *et al.* [19] studied the spatial and temporal correlations along with the collaborative nature of the wireless sensor network which can bring significant advantages for the development communication protocols. They [20] also exploited the spatial correlation on the Medium Access Control (MAC) layer. A theoretical framework was developed for transmission regulation of sensor nodes under a distortion constraint. Min *et al.* [23] considered the temporal and spatial correlations in wireless sensor networks and presented an approximate data gathering technique which is used to obtain the sensor data within the certain error bound. However, these works only considered the case that these services are fully separate and there is no correlation relationship between them.

Markov Random Field (MRF) has been successfully used to simplify many complex multi-dimension applied problems in the wireless sensor network [24–26]. Perreau *et al.* [24] presented a general framework allowing sensor networks design by using Markov Random Field (MRF) theory. They explained how the principles underlying MRF theory naturally fit to design requirements in sensor networks, especially the need to rely on distributed methods to solve global optimization problems. Wang *et al.* [25] proposed a MRF sensor fusion algorithm based on the MRF model to solve the event region detection problems. The MRF was used to model the spatial correlation. Oka *et al.* [26] presented a stochastic recursive identification algorithm which can be implemented in a distributed and scalable manner in the wireless sensor network. However, few of them are used to model the correlations of data for different services in the network in order to reduce the number of service data that needs to be reported to the application.

The node scheduling scheme is an efficient way to prolong the network lifetime [10–16]. Researchers have proposed many scheduling schemes under different application backgrounds and assumptions. Zhao *et al.* [11] presented a sleep-scheduling technique which forms multiple overlapped backbones and works alternatively. The rotation of multiple backbones contributes to balance the energy consumption of all sensor nodes, which fully utilizes the energy and extend network lifetime. Kotidis *et al.* [14] introduced the idea of snapshot queries for energy efficient data acquisition in sensor networks. They elect a small set of representative nodes in the network to constitute a network snapshot and the snapshot can be used to provide quick approximate answers to user queries. Hung *et al.* [15] proposed a centralized algorithm and a distributed algorithm to determine a set of representative nodes with high energy levels and wide data coverage ranges. Cheng *et al.*, concerned with the node scheduling problem for the service-oriented wireless sensor network [16], and proposed an Energy-aware Centralized Heuristic Scheme (ECHS) for the problem and present an Energy-aware Distributed Heuristic Scheme (EDHS) as the distributed version [9].

## 3. System Model and Problem Formulation

In this section, we firstly introduce the system model as well as some related definitions, and then formulate the multi-service data denoising problem, the representative node selection problem and the multi-service node scheduling problem. For convenience, the symbols used in this work are summarized in Table 1.

### 3.1. System Model

We consider a wireless sensor network in the plane consisting of a set of nodes *V* = {*n_i_*|1 ≤ *i* ≤ *n*}. The network needs to provide *m* sensing services *S* = {*s_i_*|1 ≤ *i* ≤ *m*}, such as temperature, sound, humidity, motion, *etc.* There is a set of targets *T* = {*t_i_*|1 ≤ *i* ≤ *k*} randomly located in the monitored area and each target needs to be provided a subset of sensing services *S*. The value of each service can be divided into several ranges according to the user's interests, such as the value of *s_i_* can be divide into *l* ranges, *i.e.*, *RA_i_* = {*ra_i_*_,_*_j_*|1 ≤ *j* ≤ *l*} = {[*a_i_*_,_*_j_*, *b_i_*_,_*_j_*]|1 ≤ *j* ≤ *l*}, and each *ra_i_*_,_*_j_* of *s_i_* has a corresponding error threshold ε*_i_*_,_*_j_* The node in the network is powered by a battery and its initial energy is denoted as *E*_0_. The node can provide multiple sensing services simultaneously, and it also knows the Euclidean distance to the sensed target, which is within its sensing radius *r* [25,26]. For conveniences, in this paper we assume that each service has an identical sensing radius and nodes only provide sensing service for one target within its sensing radius. The case that node *n_i_* provides sensing services for the *t_k_* is denoted as *pst_i_* = *t_k_* The energy cost for a node to provide each sensing service is assumed identical. Let *energy_i_* be the remained energy of node *n_i_* in current epoch and the energy cost for a node to provide a sensing service during an epoch is a constant.

Let *x_i_*_,_*_j_* represent the noise-free data sensed by node *n_i_* for *s_j_*. The sensed data *y_i_*_,_*_j_* is generally corrupted by noise in the environment as well as the sensing device, which can be formulated as:

(1) $$y_{i,j}=x_{i,j}+e_{i,j}$$

where *e_i_*_,_*_j_* is the noise and it is a Gaussian random variable which is independent and identically distributed, *i.e.*, *e_i_*_,_*_j_* ∼ *N*(0, (σ*_i_*_,_*_j_*)^2^).

For convenience, we use *X* and *Y*, respectively, to describe the noise-free data and noise-corrupted data in the network, *i.e.*, *X* = {*x_i_*_,_*_j_*|*i* ϵ *V*, *j* ϵ *S*}, *Y* = {*y_i_*_,_*_j_*|*i* ϵ *V*, *j* ϵ *S*}.

*Definition 1*: Neighbor Node Set (*NB*). The neighbor node set of node *n_i_* is denoted as *NB_i_* = {*n_j_*|*dis*(*n_i_*, *n_j_*) ≤ *r*, *pst_i_* = *pst_j_*}, where *r* is the sensing radius and *dis*(*n_i_*, *n_j_*) is the Euclidean distance between *n_i_* and *n_j_*.

Note that that node *n_i_* ϵ *NB_i_*. In this paper we assume the sensing radius of each sensing device is identical in the network. It is obvious that *n_j_* ϵ *NB_i_* in case that *n_i_* ϵ *NB_j_*. Node *n_i_* and *n_j_* are *neighbors* in this case.

*Definition 2*: Neighbor Correlated Service Set (*NS*). The neighbor correlated service set of service *s_i_* which is sensed by *n_k_* is denoted as *NS_k_*_,_*_i_* = {*s_j_*|the data for service *s_j_* and *s_i_* are correlated while *s_j_* is sensed by *n_l_* ϵ *NB_k_*}.

It is obvious that *s_i_* ϵ *NS_k_*_,_*_i_*.

*Definition 3*: Data Coverage Range (*DCR*). Given *data_i_*_,_*_j_* ϵ *ra_j_*_,_*_h_* and the error threshold ε*_j_*_,_*_h_*, the data coverage range *DCR_i_*_,_*_j_* of *data_i_*_,_*_j_* denoted as *DCR_i_*_,_*_j_* = {*data_k_*_,_*_l_*|*d*(*infer_k_*_,_*_l_*_,_*_j_*, *data_i_*_,_*_j_*) ≤ ε*_j,h_*, *n_k_* ϵ *NB_i_*, *data_i_*_,_*_j_* ϵ *ra_j_*_,_*_h_*}, where *d*(*infer_k_*_,_*_l_*_,_*_j_*, *data_i_*_,_*_j_*) = |*infer_k_*_,_*_l_*_,_*_j_* − *data_i_*_,_*_j_*|, *infer_k_*_,_*_l_*_,_*_j_* denotes that the data for *s_l_* and *s_j_* are correlated, while the data for *s_l_* in node *n_k_* inferred by the data sensed by node *n_i_* for *s_j_* is *infer_k_*_,_*_l_*_,_*_j_*.

In Definition 3, *data_k_*_,_*_l_* can be the noise-free data, noise-corrupted data or denoised data. The data *data_i_*_,_*_j_cover* any data in *DCR_i_*_,_*_j_* and each data in *DCR_i_*_,_*_j_* is *covered* by *data_i_*_,_*_j_*. It shall be mentioned that one service data sensed by a node may be in the *DCR* of the data sensed by another node for one same service, or in the *DCR* of the data sensed by the same node or the other node for different service.

*Definition 4*: Representative Node Set (*RNS*) and Their Provided Services. Given a set of sensor nodes *V*, the representative node set is a subset of *V*, and their provided services are part of the service data they sensed, and the sensed service data of each node *n_i_* ϵ *V* is either in the *RNS*'s provided service data or is in the *DCR* of *RNS*'s provided service data. Each selected node in *RNS* is one *representative node*.

Note that in this paper, the selected representative node can provide one or several service data simultaneously during the given epoch.

*Definition 5*: The *A Priori* Probability and *A Priori* Probability Distribution. For a given node *n_i_* in the network, the *a priori* probability of data *x_i_*_,_*_j_* is denoted as *P*(*x_i_*_,_*_j_*). The *A Priori* Probability Distribution *P*(*X*) is a set of *a priori* probabilities for noise-free data of nodes in the network, *i.e.*, *P*(*X*) = {*P*(*x_i_*_,_*_j_*)|*i* ϵ *V*, *j* ϵ *S*}.

*Definition 6*: Neighbor Relationship Graph (*NG*). Given a set of sensor nodes *V* and the neighbor relationship *NB*, the neighbor relationship graph of the given network is described as *NG* = (*V*, *NE*), where *NE* = {(*i*, *j*)|*n_i_* ϵ *V*, *n_j_* ϵ *NB_i_*}.

*Definition 7*: Clique. Given a neighbor relationship graph *NG* = (*V*, *NE*), a clique *c* is a subset of *V* and every two node in the subset is connected.

**Figure 3.**
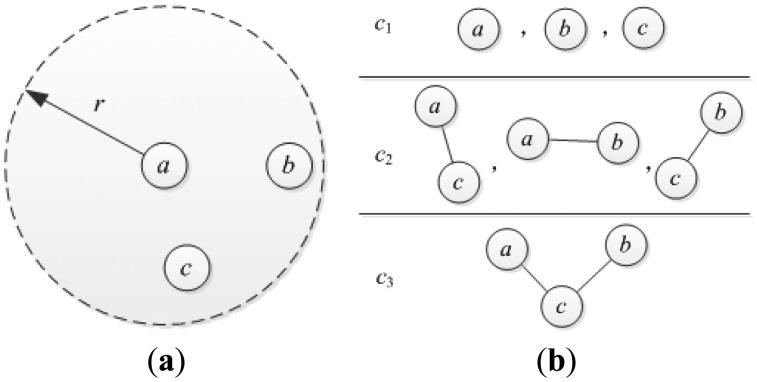
An example of clique types for a wireless sensor network. (**a**) A wireless sensor network with three nodes; (**b**) Three different clique types: single-node, pair-node and triple-node cliques.

The collection of single-node, pair-node and triple-node cliques will be denoted by *C*_1_, *C*_2_ and *C*_3_, respectively, where *C*_1_ = {(*n_i_*)|*i* ϵ *V*}, *C*_2_ = {(*n_i_, n_j_*)|*n_i_*, *n_j_* ϵ *V*, *n_j_* ϵ *NB_i_*} and *C*_3_ = {(*n_i_, n_j_*, *n_k_*)|*n_i_*, *n_j_, n_k_* ϵ *V*, *n_i_* ϵ *NB_j_*, *n_i_* ϵ *NB_k_*, *n_j_* ϵ *NB_k_*}. Figure 3 shows the clique types associated with a wireless sensor network with three nodes. The time complexity to compute all cliques for a given network is exponential to the maximum order of the cliques. It is helpful to reduce the time complexity by choosing these clique types with smaller size. In this paper, we only considered the single-node cliques *C*_1_ and pair-node cliques *C*_2_ [27].

*Definition 8*: The *A Posteriori* Probability Distribution. Given the raw noise-corrupted data *Y* as the evidence, the *a posteriori* probability distribution *P*(*X*|*Y*) is the conditional probability distribution of *X*.

### 3.2. Markov Random Field Model and Data Correlation

The Markov Random Field (MRF) model is an efficient statistical tool to describe the multi-service data distribution and correlation in multi-dimensional domains with probability functions. It can obtain global characteristics via local information and it is widely used in multi-dimensional complex applications, such as power control, event area detection and resource allocation [24–26]. MRF has two distinct characteristics when it is used in the wireless sensor networks: (1) MRF can illustrate the correlation of data for one service or two separate services in wireless sensor networks, and it also can propagate the correlation in the network which makes it possible to describe the correlation with low-order MRF; (2) MRF can reflect the uncertainty as well as the distribution of sensed multi-service data; (3) MRF model also has some other advantages such as scalability and high numerical efficiency [28].

Also, there is local correlation for data of two different services in case that the data is noise-free [14]. The correlation between the noise-free data can be expressed in form of conditional probability as shown in Formula (2):

(2) $$P(x_{i,j}|X-x_{i,j})=P(x_{i,j}|x_{NB_{i},NS_{i,j}})$$

where *X* − *x_i_*_,_*_j_* is the set difference which contains all sensed data in the network except *x_i_*_,_*_j_*, and:

(3) $$x_{NB_{i},NS_{i,j}}=\{x_{k,l}|k\in NB_{i},l\in NS_{i,j}\}$$

In Formula (2), *x_i_*_,_*_j_* is only correlated with data that belongs to *NS_i_*_,_*_j_* and is sensed by nodes in *NB_i_*. This property demonstrates that the sensed data in the multi-service wireless sensor network can be modeled with the proposed MRF model. However, it is difficult to calculate the prior probability via Formula (2). Hammersley–Clifford Theorem shows that MRF is equivalent to Gibbs Random Field (GRF) [27]. It means the conditional probability of MRF can be obtained with GRF.

The GRF can be built with the correlation probability distribution of the noise-free data *X* as follows:

(4) $$P\left(X\right)=\frac{1}{Z}exp^{U\left(X\right)}$$

where $U\left(X\right)=\sum_{i\in V}V_{1}\left(x_{i}\right)+\sum_{i\in V}\sum_{j\in N_{i}}V_{2}(x_{i},x_{j})$is the prior energy of *X*, *V*_1_(*x_i_*) and *V*_2_(*x_i_*, *x_j_*) are the potential functions for cliques in *C*_1_ and *C*_2_ respectively, and *Z* is a normalizing constant used as the partition function.

Now consider the noise version of the sensed data *y_i_*_,_*_j_* compared with the noise-free data *x_i_*_,_*_j_*. The conditional probability for *Y* is formulated as:

(5) $$p(Y|X)=\frac{1}{\Pi_{i=1}^{n\times m}\sqrt{2\pi \sigma^{2}}}e^{-U(Y|X)}$$

where *U*(*Y*|*X*) is the likelihood energy, *n* × *m* is the number of sensed data in the network.

There is a negative correlation between the posterior probability and the likelihood energy of sensed data, and we have the correlation with Formula (6):

(6) $$P(X|Y)\propto e^{-U(X|Y)}$$

where:

(7) U(X|Y)=U(Y|X)+U(X)

As we can see from Formulas (6) and (7), GRF is helpful to obtain the posterior probability *P*(*X*|*Y*) in MRF, and it provides a simple method to calculate the joint probability *P*(*X*|*Y*) by specifying the clique potential functions and choosing the proper potential functions for desired correlated system.

### 3.3. Multi-Service Data Denoising Problem

The multi-service data denoising problem concerns with the issue of minimizing the noise level of sensed data by utilizing the correlation of data for multiple services. As described in the introduction, this problem is helpful to reduce the number of service data which is reported to the application. One intuitive method for the multi-service data denoising problem is to obtain the optimal data *Y*′ by minimizing the conational probability as follows:

(8) $$Y'=\underset{Y'}{\arg\min}P(Y'|Y)$$

According to Formula (6), the object of the multi-service data denoising problem is to minimize the likelihood energy as follows:

(9) $$Y'=\underset{Y'}{\arg\min}U(Y'|Y)$$

There is Markov dependence in the multi-service sensed data due to the spatial continuous variation and multi-service correlation in the practical environment [19,23]. To calculate the optimal data *Y′* in Formula (9), we adopt the clique potential functions *U*(*Y′*|*Y*) = *U*(*Y′*) + *U*(*Y*|*Y′*) as follows:

(1) *U*(*Y′*) is used to present the correlation between data for one single service. The data sensed by one node for a service is correlated with data sensed by adjacent nodes for the same service. Thus we have Formula (10):
  (10) $$U(Y')=\sum_{k\in S}\sum_{i\in V}V_{1}(y_{i,k}^{'})+\sum_{k\in S}\sum_{i\in V}\sum_{j\in NB_{i}}V_{2}(y_{i,k}^{'},y_{j,k}^{'})$$
  where $V_{1}\left(y_{i,k}^{'}\right)={\left(y_{i,k}^{'}-y_{i,k}\right)}^{2}$ and $V_{2}\left(y_{i,k}^{'},y_{j,k}^{'}\right)={\left(y_{i,k}^{'}-y_{j,k}^{'}\right)}^{2}$
*U*(*Y*|*X*) is used to present the correlation between data for different services. The service data sensed by one node is correlated with data sensed by adjacent nodes for another service because there is correlation between two different services. Thus we have Formula (11):
  (11) $$U(Y|Y')=\sum_{l\in NS_{i,k}}\sum_{k\in S}\sum_{i\in V}V_{1}(y_{i,k}|y_{i,l}^{'})+\sum_{l\in NS_{i,k}}\sum_{k\in S}\sum_{i\in V}\sum_{j\in NB_{i}}V_{2}(y_{i,k}|y_{i,k}^{'},y_{j,l}|y_{j,l}^{'})$$
  where $V_{1}\left(y_{i,k}^{'}|y_{i,l}\right)={\left(y_{i,k}^{'}-\text{infe}r_{i,j,k}\right)}^{2}$ and $V_{1}\left(y_{i,k}|y_{i,k}^{'},y_{j,l}|y_{j,l}^{'}\right)={\left(y_{i,k}^{'}-{\text{infe}}_{j,l,k}\right)}^{2}$
  Therefore, the likelihood energy is described as follows:
  (12) $$\begin{array}{ll} U(Y'|Y)= & \sum_{k\in S}\sum_{i\in V}{\left(y_{i,k}^{'}-y_{i,k}\right)}^{2}+\sum_{k\in S}\sum_{i\in V}\sum_{j\in NB_{i}}{\left(y_{i,k}^{'}-y_{j,k}^{'}\right)}^{2}\\ & +\sum_{l\in NS_{i,k}}\sum_{k\in S}\sum_{i\in V}{\left(y_{i,k}^{'}-infer_{i,l,k}\right)}^{2}+\sum_{l\in NS_{i,k}}\sum_{k\in S}\sum_{i\in V}\sum_{j\in NB_{i}}{\left(y_{i,k}^{'}-infer_{j,l,k}\right)}^{2} \end{array}$$

According to Formula (12), the likelihood energy of a single service data *y′_i_*_,_ *_j_* is deduced as follows:

(13) $$\begin{array}{ll} U(y_{i,j}^{\prime }|y_{NB_{i},NS_{i,j}})= & {\left(y_{i,j}^{'}-y_{i,j}^{'}\right)}^{2}+\sum_{k\in NB_{i,j}}{\left(y_{i,j}^{'}-y_{k,j}^{'}\right)}^{2}\\ & +\sum_{l\in NS_{i,j}}{\left(y_{i,j}^{'}-\text{infe}r_{i,l,j}\right)}^{2}+\sum_{l\in NS_{i,j}}\sum_{k\in NB_{i}}{\left(y_{i,j}^{'}-infer_{k,l,j}\right)}^{2} \end{array}$$

### 3.4. Representative Node Selection Problem

During each epoch, a set of representative nodes are selected and their provided services are also determined, and they can cover the data of other nodes in the network within the given error threshold. Note that the representative nodes should retain with high residual energy so that they can provide the determined services during the given epoch.

*Definition 9*: Representative Node Selection Problem. Given a set of sensor nodes *V*, the neighbor relationship graph *NG* = (*V*, *NE*), the denoised data *Y′*, {*energy_i_*|*i* ϵ *V*}, error threshold {ε*_i_*_,_ *_j_*|*i* ϵ *S*, *j* ϵ *ra_i_*} and the correlation of data for multiple services, the representative node selection problem is to select a number of representative nodes and determine their provided services to minimize the energy cost during a given epoch, and ensure the selected representative nodes with high residual energy.

### 3.5. Multi-Service Node Scheduling Problem

The network lifetime is one of the most important metrics for the wireless sensor networks, and there are various measurements for it, such as the first node to die, the number of survived nodes, and the fraction of survived nodes [29]. Due to the fact that the sensor network is generally densely deployed, the network can still keep on providing the required services although the first node is dead. In this paper, we use the fraction of survived nodes to measure the network lifetime. Let *SN*(*t*) denote the number of nodes that are survived to provide services at time slot *t*, the network lifetime *TL* is defined as follows:

(14) TL=max{t:SN(t)≥τ×SN(0)},

where *τ* denotes a given survived nodes threshold.

It is obvious that the network lifetime indicates the time period during which the wireless sensor network can keep on providing the required multiple services. The wireless sensor network collects data in a periodical manner, and the time duration to collect all service data to the application is called an epoch.

The representative node selection problem aims at reducing the number of service data that is reported to the application and minimizing the energy cost during a given epoch. However, these selected representative nodes might deplete the energy if they are always keep on working and thus the network will rapidly run to death. The multi-service node scheduling problem is to select a set of nodes and determine the services they provided in a periodical manner [30]. When the timer expires, a new set of nodes is selected and their provided services are also determined. In this way, the energy consumption keeps balanced in the network. This method is helpful to enlarge the network lifetime especially in densely deployed wireless sensor networks in which network lifetime is one of the most important metrics [31]. The definition of the problem is shown as follows.

*Definition 10*: Multi-service Node Scheduling Problem. Given a set of sensor node *V*, sensing radius *r*, the noise-corrupted data *Y* = {*y_i_*_,_*_j_*|*i* ϵ *V*, *j* ϵ *S*}, error threshold {ε*_i_*_,_*_j_*|*i* ϵ *S*, *j* ϵ *ra_i_*}, {*energy_i_*|*i* ϵ *V*} and the correlation between different services, the Multi-service Node Scheduling Problem is to select a set of representative nodes and determine their provided services in a periodical way and aim at maximizing the network lifetime *TL*.

## 4. Proposed Scheme

As described in Section 2, the sensed data is correlation and redundancy occurs in the network. The node scheduling scheme is an important way to solve the problem of resource sharing with different services. At the same time, an efficient data denoising algorithm before the node scheduling can efficiently reduce the number of data that is sent to the application during a given epoch. In this section, we firstly propose a Multi-service Data Denoising (MDD) algorithm for the multi-service data denoising problem; Secondly, we introduce the multi-service Representative node Selection and service Determination (RSD) algorithm for the problem in Definition 9; Finally, the MRF-based Multi-service Node Scheduling (MMNS) scheme is proposed to solve the problem in Definition 10.

### 4.1. Multi-Service Data Denoising (MDD) Algorithm

The multi-service data denoising problem defined in Formula (9) is an un-constrained optimization problem. In this paper, we propose a Multi-service Data Denoising (MDD) algorithm to solve the problem by referring to the Iterative Conditional Modes (ICM) algorithm [27]. The ICM algorithm uses a “greedy” strategy in the iterative local maximization: Given the data *Y*, the ICM algorithm sequentially updates each *Y*′*_i_^k^* into *Y*′*_i_*^(^*^k^*^+1)^ by maximizing *P(Y*′|*Y*), where *k* denotes the *k*-th iteration. Due to the negative correlation between the posterior probability and the likelihood energy defined in Formula (6), we sequentially denoise the selected sensed data by minimizing *U*(*y*′*_i_*_,_*_j_*|*y_NBi_*_,_ *_NSi_*_,_ *_j_*) which is defined in Formula (13).

In Formula (13), the likelihood energy function of node *n_j_* is a quadratic function and there is only one minimum value in the real number range. We calculate the extremum of *U*(*y′_i_*_,_*_j_*|*y_NBi_*_,_ *_NSi_*_,_ *_j_*) in real number range with Formula (16) when derivative of *U*(*y′_i_*_,_*_j_*|*y_NBi_*_,_ *_NSi_*_,_ *_j_*) in Formula (15) is zero:

(15) $$\frac{\partial U(y_{i,j}^{\prime }|y_{NB_{i},NS_{i,j}})}{\partial y_{i,j}^{\prime }}=0$$

Then we have the optimal *opt*(*y*′*_i_*_,_ *_j_*) as follows:

(16) $$opt(y_{i,j}^{\prime })=\frac{\left(y_{i,k}+\sum_{j\in N_{i}}y_{j,k})+(infer_{i,l,k}+\sum_{j\in N_{i}}infer_{j,l,k}\right)}{2\times (1+\sum_{i\in N_{i}}1)}$$

**Algorithm 1. Multi-service Data Denoising (MDD) algorithm.**
**Input: *NG*** = **(*Temp*, *NE*), *Y*** = {***y****_i_***_,_**
*_j_***|*i* ϵ *Temp*, *j* ϵ *S*},** {**ε***_i_***_,_**
*_j_***|*i* ϵ *S*, *j* ϵ *ra****_i_***},** {***energy****_i_***|*i* ϵ *Temp*}, the Correlation of Data for Multiple Services;**
**Output: *Y*′** = {***y*′***_i_***_,_**
*_j_***, *i* ϵ *Temp*, *j* ϵ *S*}**
1. Set the state of all service data sensed by nodes in *Temp* as *UN-DENOISED*;2. *Change* = 1;3. While *Change*4. *Change* = 0;5. For each node *n_i_* in *Temp*6. For each *UN- DENOISED* service data *y_i_*_,_
*_j_* sensed by node *n_i_*7. Calculate the optimal *opt*(*y*′*_i_*_,_
*_j_*) with Formula (16);8. If *opt*(*y*′*_i_*_,_
*_j_*) ≠ *y_i_*_,_
*_j_* then9. *y_i_*_,_
*_j_* ← *opt*(*y*′*_i_*_,_
*_j_*); *Change* = 1; Set the state of *y_i_*_,_
*_j_* as *DENOISED*;10. End if11. End for12. If the state of all service data sensed by node *n_i_* has been marked as *DENOISED*, then13. *Temp* ←*Temp* − {*n_i_*};14. End if15. End for16. End while

As we can see from Algorithm 1, the proposed MDD algorithm is carried out in an iterative manner. Each survived node *n_j_* ϵ *Temp* checks its *UN-DENOISED* service data iteratively to ensure a local maximization of conditional probability. This check process is carried out with a local variable *Change* which is initialized as 1 (Line 2). The *Change* is set as 0 if no sensed dada can be denoised (Line 4). If one data can be denoised, the optimal value *opt*(*y*′*_j_*) is calculated with Formula (16) (Line 7). Then we denoise *y_i_*_,_*_j_* with *opt*(*y*′*_i_*_,_*_j_*) and set *Change* to 1 in case that *opt*(*y*′*_i_*_,_*_j_*) is not equal to *y_i_*_,_*_j_* (Line 8–10). In this way, we obtain the approximate optimal solution for the multi-service data denoising problem by greedily calculating the optimal value for all service data of *n_j_* ϵ *Temp*.

### 4.2. Multi-Service Representative Node Selection and Service Determination (RSD) Algorithm

In this section, we introduce a novel multi-service Representative node Selection and service Determination (RSD) algorithm by using the correlation of data for multiple services. The multi-service representative node selection problem in Definition 9 is also a multi-objective nonlinear optimization problem. There are already several solutions to this kind of problems for the single service wireless sensor networks [14,15]. In the service-oriented wireless sensor network, nodes can support multiple sensing services rather than one single service and these sensing services are correlated. How to solve the problem of resources sharing and provide all these different services in an energy-efficient way is an important problem in the wireless sensor network.


**Algorithm 2.** Multi-service Representative node selection and Service Determination (RSD) algorithm.
**Input: *Temp*, *Y′*** = {***y′****_i_***_,_**
*_j_***|*i* ϵ *Temp*, *j* ϵ *S*}, *NG*** = **(*V*, *NE*),** {***energy****_i_***|*i* ϵ *Temp* },** {***ε****_i_***_,_**
*_j_***|*i* ϵ *S*, *j* ϵ *ra****_i_***}, the correlation of data for multiple services;**
**Output: *RNS* and their provided services *TPS***.
1. Calculate *DCR_i_*_,_
*_j_* = {*j*|*d*(*infer_i_*_,_
*_j_*_,_
*_l_*, *y′_k_*_,_
*_l_*) ≤ ε*_l, h_*, *n_j_* ϵ *NB_i_*, *y′_k_*_,_
*_l_* ϵ *ra_l, h_*}, *i* ϵ *V*, *j* ϵ *S*;2. *RNS* ← Ø; *TPS* ← Ø;3. Mark all service data sensed by nodes in *Temp* as *UN-COVERED*;4. While there is *UN-COVERED* service data sensed by nodes in *Temp*5. Select service data *y′_i_*_,_
*_j_* with maximum *DCR* size in the network; if there are several service data with the same maximum *DCR*, select the one with maximum residual energy;6. Recalculate *DCR_i_*_,_
*_j_* for each service data *y′_i_*_,_
*_j_* of node *n_i_* ϵ *Temp*;7. *RNS* ← *RNS* + {*i*}; *TPS* ← *TPS* + { *y′_i_*_,_
*_j_*};8. Mark the *j*-th service of node *n_i_* and all service data in *DCR_i_*_,_
*_j_* as *COVERED*;9. If all service data sensed by node *n_i_* is marked as *COVERED*, then *Temp* ← *Temp* − *DCR_i_* − {*i*};10. End while

We adopt a greedy strategy to select the representative nodes and determine the services they provided. Initially, we calculate the *DCR* of each service data and mark the state of all service data as the state of *UN-COVERED*; Then, we select a service data with maximum *DCR* in a sequent way. The selected service data and the data in the *DCR* are marked as *COVERED*. Repeat this process until all service data are marked as *COVERED*. The pseudo code for RSD is shown in Algorithm 2. The process is carried out as follows. Firstly, we calculate *DCR_i_*_,_*_j_* for each denoised data *y′_i_*_,_*_j_* sensed by node *n_i_* in the networks (Line 1). Then, initialize the representative node set *RNS* and their provided services *TPS* as empty set (Line 2). Thirdly, mark all service data sensed by nodes in *Temp* as *UN-COVERED* (Line 3). Finally, we select the representative node *n_i_* and its provided data of *s_j_* in an iterative manner in which the data of *s_j_* sensed by node *n_i_* can cover the maximum number of *UN-COVERED* service data in the network (Line 5–9). In case that a service data sensed by one node is already provided, the service data and all service data in its data coverage range are marked as *COVERED* (Line 8). In Line 9, in case that all service data sensed by one node is marked as *COVERED*, the node is removed from *Temp*. This process continues until the state of all service data is *COVERED* (Line 4–10).

### 4.3. MRF-Based Multi-Service Node Scheduling (MMNS) Scheme

The multi-service node scheduling problem in Definition 10 is NP-hard and a heuristic solution is generally an efficient approach to solve it [14]. As shown in Section 1, nodes scheduling during a given epoch can not only reduce the number of service data that is reported to the application and thus prolong network lifetime, but also contribute to solve some other issues in densely deployed wireless sensor networks. However, the selected *RNS* and their provided services should be reselected in a periodical manner: (1) The representative nodes need to report their provided service data, and thus they consume more energy than other nodes in the network, which means that the representative nodes should be reselected when they have less residual energy to avoid node failure; (2) The selected service data provided by representative nodes may not cover those service data in their *DCR* due to environmental changes; (3) The *RNS* and their provided services should be reselected in a periodical in order to balance the energy consumption in the network and thus prolong the network lifetime [30,31].

The work proposed by [14,15] generally intended to select the representative nodes with the raw noise-corrupted data and ignored the noise in the raw data. As shown in Section 1, it is not an efficient way to select the representative nodes and determine the services they provided by raw noise-corrupted data. We introduce to denoise the sensed multi-service data before scheduling representative nodes and their provided services during a given epoch in the proposed scheduling scheme.


**Algorithm 3. MRF-based Multi-service Node Scheduling (MMNS) scheme.**
**Input: *V*, *r*, *Y*** = {***y****_i_***_,_**
*_j_***|*i* ϵ *V*, *j* ϵ *S*},** {***ε****_i_***_,_***_j_***|*i* ϵ *S*, *j* ϵ *ra****_i_***},** {***energy****_i_***|*i* ϵ *V*}, the correlation between different services;****Output: Scheduling schemes.**1. *Temp* ← *V* − {*n_j_* | *energy_i_* ≤ 0, *i* ϵ *V*};2. Calculate *NB_i_* for node *n_i_* ϵ *Temp* through information exchange;3. Construct neighbor relationship graph *NG*;4. Model correlation of data for multiple services with the MRF model;5. Run MDD algorithm to denoise the sensed service data in the network;6. Run RSD algorithm to get the *RNS* and the provided services;7. Nodes in *RNS* collect and send their provided service data to the application during current epoch;8. Calculate the residual energy of nodes as well as *SN*(*t*), if *SN*(*t*) ≥ *τ* × *SN*(0) is not satisfied, go to Step 10;9. Go to Step 1 and start one new epoch;10. End.

Algorithm 3 lists the pseudo code for the proposed MMNS. Firstly, we remove the dead node in the network and construct *NB_i_* for each node *n_i_* in the network through information exchange (Line 1–2). The neighbor relationship graph *NG* is built in Line 3. In Line 4, the correlation of data for multiple services is modeled with the proposed MRF model. Then, run MDD algorithm to denoise the sensed multi-service data (Line 5). The RSD algorithm is carried out to get a *RNS* and their provided services (Line 6). Nodes in *RNS* report their provided service data to application during the current epoch (Line 7). In Line 8, the residual energy of nodes in the network is calculated as well as *SN*(*t*); and then check if the network dead or not after collecting sensed service data provided by *RNS* during the epoch, and go to Step 10 if the network dead (Line 8); otherwise, go to Step 1 to get a new *RNS* and determine their provided services (Line 9). Repeat this process until the network is dead, *i.e.*, *SN*(*t*) < *τ* × *SN*(0).

## 5. Time Complexity Analysis

*Theorem 1*. The time complexity of MDP algorithm is *O*(*n*^2^ × *k*^2^), where *k* denotes the number of services in the network.

Proof: The time complexity of setting the state of all service data sensed by nodes in *Temp* with state as *UN-DENOISED* is *O*(*n* × *k*), where *k* denotes the number of services in the network. The time complexity of selecting a service data with maximum *DCR* is *O*(*n* × *k*), and this process runs at most *O*(*n* × *k*) times. The time complexity of calculating the *opt*(*y*′*_i_*_,_*_j_*) is *O*(*k* × *n_max*), and the is process runs at most *O*(*n* × *k*) times, where *n_max* denotes the maximum number of nodes in the neighbor node set and *n_max* ≤ *n*. The time complexity of checking the state of all service data provided by a given node is *O*(*k*) and the time complexity of checking the state of all service data provided by all nodes is *O*(*n* × *k*).

Thus, the time complexity of MDP algorithm is *O*(*n* × *k*) + *O*(*n*^2^ × *k*^2^) + *O*(*n* × *k*^2^ × *n_max*) + *O*(*n* × *k*) = *O*(*n*^2^ × *k*^2^).

*Theorem 2*. The time complexity of RSD algorithm is *O*(*n_max* × *k*^2^ × *n*^2^), where *n_max* denotes the maximum number of nodes in the neighbor node set and *k* denotes the number of services in the network.

Proof: It is easy to know that the time complexity of calculating *DCR* for all service data in the network is *O*(*n_max* × *n* × *k*) and the process runs at most *O*(*n* × *k*) times. The time complexity of marking all service data sensed by nodes in *Temp* with state as *UN-COVERED* is *O*(*n* × *k*). The time complexity of selecting the *RNS* as well as determining their provided services is *O*(*n*^2^ × *k*^2^): The time complexity of selecting a service data with maximal *DCR* is O(*n* × *k*) and the process runs at most O(*n* × *k*) times.

In this way, the time complexity of RSD algorithm is *O*(*n_max* × *n*^2^ × *k*^2^) + *O*(*n* × *k*) + *O*(*n*^2^ × *k*^2^) = *O*(*n_max* × *n*^2^ × *k*^2^).

## 6. Simulation Results and Analysis

In this section, we evaluate the performance of our proposed scheme by demonstrating detailed simulation experiments. The related works [14,15] are most close to the problems we studied in this paper. Kotidis [14] proposed to select the representative nodes with the partially ordered tuple <data coverage range, residual energy>. Peng *et al.* [15] proposed to use the partially ordered tuple <residual energy, data coverage range>. Both of them do not consider the correlation of data for different services and thus they cannot be directly used to our problems in this paper.

Here we modify the above two schemes for possible comparisons: (1) The first scheme named as Multi-service Node Scheduling (MNS) scheme which is originated from [14]. MNS uses the partially ordered tuple <*DCR*, residual energy> to schedule node and determine the services they provided, where the definition of data coverage range is the same as that in Definition 3, but no denoising algorithm is applied before scheduling; (2) The second algorithm named the Energy priority-based multi-service Node Scheduling (ENS) scheme is originated from [15]. ENS uses the partially ordered tuple <residual energy, data coverage range> and selects the node with maximum residual energy and all its sensed service data will be provided.

In addition, we design another scheme named Single service Node Scheduling (SNS) scheme for further comparison, which only considers the correlation for data of one service, but not correlation of data for two separate services. SNS adopts the partially ordered tuple <data coverage range, residual energy> to schedule nodes for each service independently, and the data coverage range only considers one single service. Then, in the simulation we compare the proposed MMNS scheme with MNS, ENS and SNS by running them in the same networks with same parameters in the environments.

Here we adopt two main metrics to compare the performance, *i.e.*, the energy cost during a given epoch and the network lifetime. The energy cost during a given epoch is an important measurement metric since energy cost during a given epoch denotes fewer service data. Meanwhile, the network lifetime is one main designing goal for wireless sensor networks [1].

### 6.1. Simulation Environments

We adopt Matlab as the platform tool which is widely used in the simulation experiments of wireless sensor networks. The default experimental parameters are set as follows: nodes and targets are placed in a randomly manner over the monitored area. For convenience, we assume that each service data is transformed into the same value range and the initial value of each service data is randomly selected from [0, 50]. The value range of each service can be divided into three sub-ranges, *i.e.*, [0, 10], [10, 40] and [40, 50]. The default values of the simulation parameters are shown in Table 2. The demonstrated metrics are calculated by averaging the results of 10 different network topologies.

We adopt the method of generating synthetic sensed data on the monitored area. In the synthetic data set, each service is randomly generate in every target with probability *sgp* = 0.6. One important issue is how to simulate the correlation of data for multiple services in wireless sensor networks. The basic idea is described as follows. Firstly, we generate the correlation of data for these five services. If there are two service data in a target falling into different ranges of the three ranges: *ra*_1, 2_, *ra*_2, 2_ and *ra*_3, 2_, then it is said that these two service data in the target can infer each other. If there are two service data in a target falling into different ranges of *ra*_4, 1_ and *ra*_5, 1_, then it is said that these two data can also infer each other. For example, there are two service data in a target, the first service data in the target is 20 and it is falling into *ra*_1,2_, and the second data falling into *ra*_2, 2_. Then, we can infer that the value of the second data in the target is also 20.

Then, we generate the service data in each target in a sequence way when the current service data has a correlation with any other service data. For example, we assume that a target need to generate the former two of the five service data, and the data for *s*_1_ generate randomly, then the data for *s*_2_ generates its data according to the data for *s*_1_.

Each node in the network equipped with five sensing devices and can sense those service data at the target. The sensed service data for a given node is produced accordingly to the nearest target within its sensing radius. The deviation σ*_i_*_,_*_j_* between the actual sensed data and the real service data at the target is proportional to their Euclidean distance [28]. The value of σ*_i_*_,_*_j_* is shown in Formula (17) [19]:

(17) $$\sigma_{i,j}=\left\{ \begin{array}{l} \frac{3}{2}\frac{d(n_{i},t_{k})}{r}-\frac{1}{2}{\left(\frac{d(n_{i},t_{k})}{r}\right)}^{3},\;\text{if}\;0\leq \;dis(n_{i},t_{k})\leq r\\ 0,\;\text{if}\;dis\;(n_{i},t_{k})>r \end{array}\right.$$

Here, *target_k_*_,_ *_j_* denotes the data for service *s_j_* in target *t_k_*. The value of each noise-corrupted service data sensed by a given node which monitors *t_k_* is simulated as follows:

(18) $$y_{i,j}=x_{i,j}+e_{i,j}=target_{k,j}+e_{i,j},$$

where *e_i_*_,_ *_j_* ∼ *N*(0, (σ*_i_*_,_*_j_*)^2^).

The value of each service data in a target changes over time with the value formulated as:

(19) $$target_{i,j}\left(t\right)=target_{i,j}\left(t-interval\right)+Z,$$

where *interval* is the collection time of sensed data from all nodes to the application during an epoch, and *Z* is a random variable that satisfies the Gaussian random distribution with mean as 0 and variance as 0.5.

### 6.2. Noise Level of Denoised Data

As shown in Formula (20), we adopt the Sum of Square of Deviations (*SSD*) as the metric to compare the noise level between the raw noise-corrupted and denoised data in this paper:

(20) $$SSD(Y\prime ,X\text{)}=\sum_{i=1}^{n}\sum_{j=1}^{m}||y_{i,j}^{\prime }-x_{i,j}|{|}^{2}$$

The simulation result is demonstrated in Table 3. The number of nodes in the network is set varying from 100 to 500 with an increment of 100. As we can see, the noise of raw noise-corrupted data has a positive correlation with the number of nodes in the network, while the noise of denoised data is independent. The noise of denoised data is always smaller than that of raw noise-corrupted data with the incensement of network size. It means that the proposed scheme can efficiently enlarge the network lifetime without increasing the noise of collected data.

### 6.3. Comparison of Energy Cost

In this section, we compare the energy cost of the selected representative nodes to provide their determined sensing services during a given epoch with the proposed MMNS, MNS, ENS and SNS via various parameters, including error threshold, network size and number of targets.

#### 6.3.1. Impact of Parameter ε_1_

**Figure 4.**
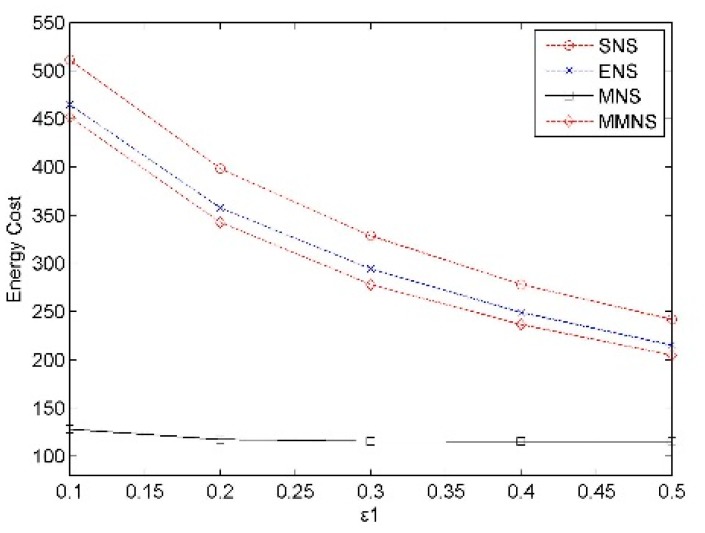
The impact of the ε_1_ on the energy cost.

In the simulation, the error threshold ε_1_ varies from 0.1 to 0.5 with an increment as 0.1. The error threshold ε_2_ is double of ε_1_, while ε_3_ is equal to ε_1_. Intuitively, the increasing of error threshold demonstrates that more data deviations are tolerant in the network and the less energy cost is necessary to provide the sensing services during a given epoch. In Figure 4, the energy cost decreases obviously with the error threshold increasing with schemes SNS, ENS and MNS. This is reasonable because the size of *DCR* increases too with the error threshold increasing. However, this trend is not so significant with MMNS, because our denoising algorithm can efficiently enlarge the size of *DCR* when the error threshold is small. In case that ε_1_ = 0.3, the energy cost of MMNS is about 35.1%, 39.2%, and 41.5% of that of SNS, ENS and MNS. It shows that our proposed denoising algorithm can *significantly* reduce the energy cost by utilizing the correlation with the MRF model. More importantly, the proposed scheme runs stable compared with related schemes when ε_1_ varies from 0.1 to 0.5.

#### 6.3.2. Impact of Network Size

**Figure 5.**
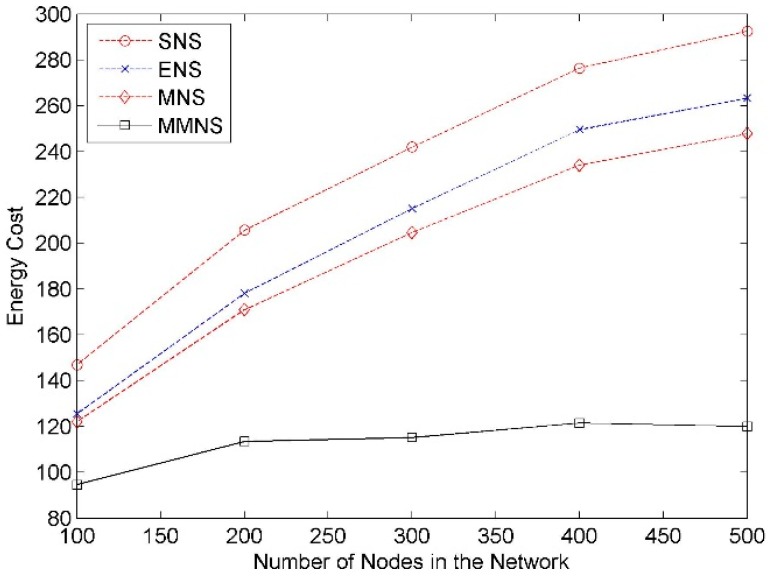
The impact of the network size on the energy cost.

The network size varies from 100 to 500 with an increment as 100. The simulation result is demonstrated in Figure 5. It shows that the energy cost ascends with network size increasing. However, this trend is not obvious when the network size is larger than a given point, *i.e.*, 300. It is due to the fact that the services provided by a given set of representative nodes can cover all service data sensed by nodes in a network when the network is densely deployed. The new added nodes and their sensed service data is generally covered by the current selected representative nodes. In all cases, our proposed scheme always has better performance compared with the other three schemes. In addition, the size of energy cost keeps stable compared with the increment of nodes in the network.

#### 6.3.3. Impact of Number of Targets in the Network

**Figure 6.**
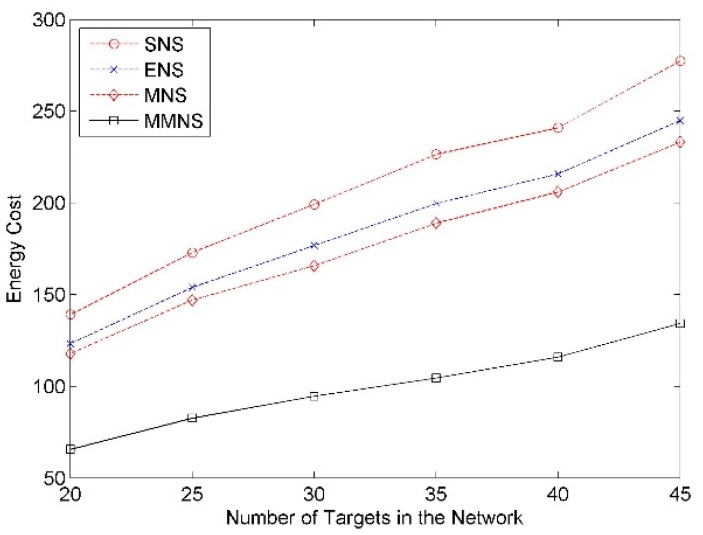
The impact of the number of targets on the energy cost.

The number of targets varies from 20 to 45 with an increment as 5 and the simulation result is demonstrated in Figure 6. It shows that the energy cost increases with the number of targets. It can also be seen that MMNS has better performance compared with the other three schemes regardless of the number of events. This trend is more obvious as the number of targets increases.

### 6.4. Comparison of Network Lifetime

In this section, we compare the network lifetime of proposed MMNS with MNS, ENS and SNS by various parameters, including the error threshold, network size and number of targets.

#### 6.4.1. Impact of Error Threshold ε_1_

**Figure 7.**
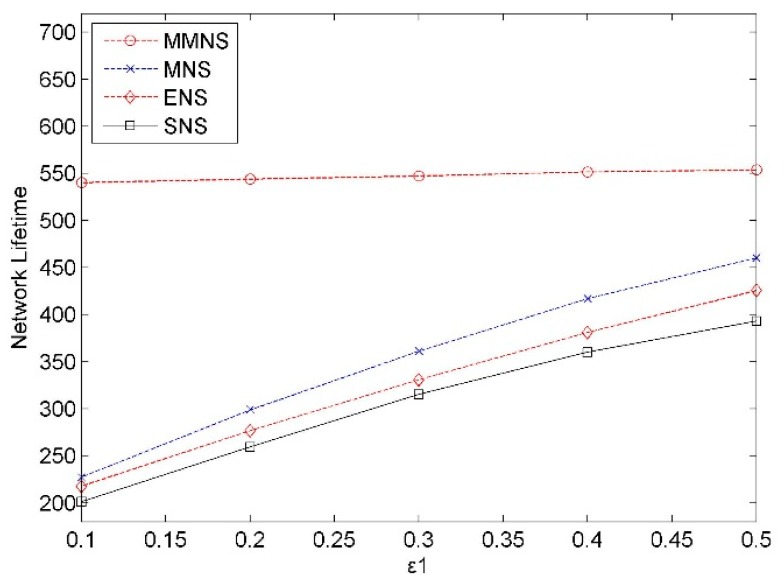
The impact of the error threshold on the network lifetime.

The error threshold varies from 0.1 to 0.5 with an increment as 0.1 in the simulations. As shown in Figure 7, the network lifetime increases along with the error threshold. In all cases, the network lifetime with MMNS is always longer than the other three schemes. For example, in case ε_1_ = 0.3, there is about 51.5%, 65.4% and 73.5% increment by comparing MMNS with MNS, ENS and SNS.

#### 6.4.2. Impact of Network Size

**Figure 8.**
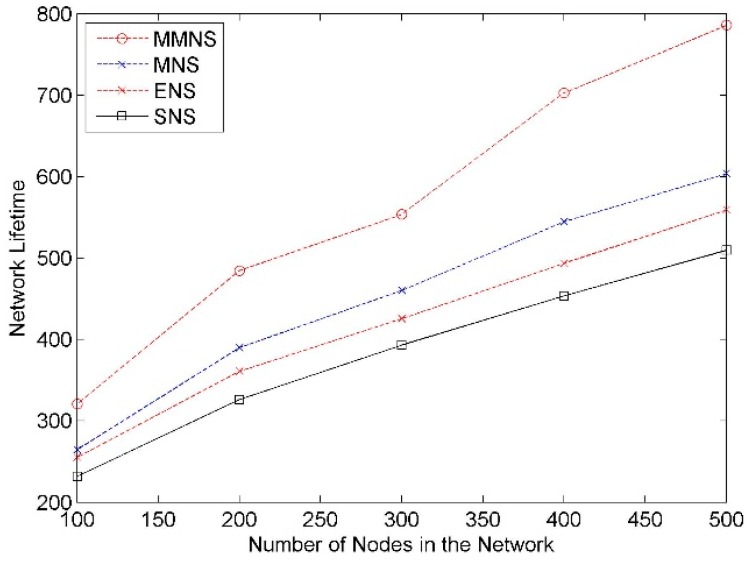
The impact of the network size on the network lifetime.

The network size is set from 100 to 500 with an increment as 100, and the result is demonstrated in Figure 8. It shows that the network lifetime increases along with the network size. This is reasonable because the new added nodes are helpful to increase the redundancy in the network and extend the network lifetime accordingly. As we can see from Figure 8, MMNS has better performance compared with related schemes regardless of network size in all cases. For example, in case *n* = 300, there is about 20.3%, 30.1% and 40.8% increment by comparing MMNS with MNS, ENS and SNS.

#### 6.4.3. Impact of Number of Targets

**Figure 9.**
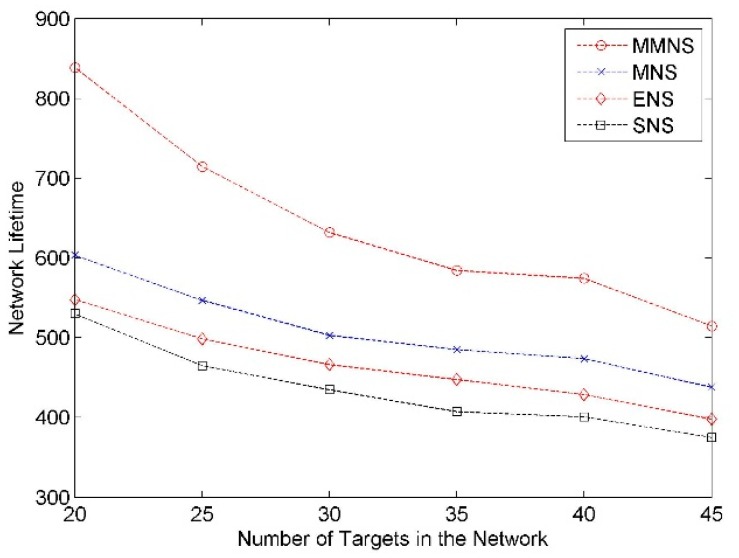
The impact of the number of targets on the network lifetime.

The number of targets varies from 20 to 45 with an increment as 5 and the simulation result is demonstrated in Figure 9. It shows that the energy cost increases with the number of targets by using the correlation data generation process. It can be seen that MMNS has better performance compared with the other three schemes regardless of the number of events.

## 7. Conclusions

Energy-efficient multi-service node scheduling is an important issue in a wireless sensor network by exploiting the data correlation of data for multiple services. In this paper, we introduce the Markov Random Field (MRF) model to describe these correlations in the network. The raw noise-corrupted data is also discussed and the multi-service data denoising (MDD) algorithm is proposed to minimize the noise level of the sensed multi-service data. We also propose the multi-service Representative node Selection and service Determination (RSD) algorithm and MRF-based Multi-service Node Scheduling (MMNS), respectively, for the representative node selection and their provided Service Determination problem and the multi-service node scheduling problem. Experimental results on synthesized data sets show that RSD can obviously reduce the energy cost during a given epoch, and MMNS can obviously extend the network lifetime compared with related schemes. In future work, we will further consider the temporal correlation in the network and design an efficient multi-service node scheduling scheme with both spatial and temporal correlation considered.

## Figures and Tables

**Table 1.** Notation of the symbols.

**Symbol**	**Description**
*n_i_*	**The *i*-th node in the network**
*s_i_*	**The *i*-th service in the network**
*t_i_*	**The *i*-th target in the network**
*ra_i_*_,_ *_j_*	**The *j*-th value range of *s****_i_*
ε*_i, j_*	**Error threshold for the value range *ra****_i_* **_,_***_j_* **of *s****_i_*
*pst_i_*	**Target monitored by node *n****_i_*
*r*	**Sensing radius**
*energy_i_*	**Residual energy of node *n****_i_*
*x_i_*_,_ *_j_*, *y_i_*_,_ *_j_*, *y′_i_*_,_ *_j_*	**Noise-free, Noise-corrupted and Denoised data of *s****_j_* **sensed by node *n****_i_*
*e_i_*_,_ *_j_*	**Noise in the data of *y****_i_***_,_** *_j_* **node *n****_i_*
*NB_i_*	**Neighbor node set of node *n****_i_*
*NS_k_*_,_ *_i_*	**Neighbor correlated service set of *s****_i_* **sensed by node *n****_k_*
*DCR_i_*_,_ *_j_*	**Data coverage range of the data of *s****_j_* **sensed by node *n****_i_*
*infer_k_*_,_ *_l_*_,_ *_j_*	**Data for service *s****_j_* **and *s****_l_* **are correlated, and the data for *s****_l_* **in node *n****_k_* **inferred by the data of *s****_j_* **sensed by the other node is *infer****_k_***_,_** *_l_***_,_** *_j_*

**Table 2.** Default values for the simulation parameters.

**Parameter Description**	**Default Value**
Monitored area size	100 m × 100 m
Number of nodes in the network	300
Sensing radius	10 m
Number of targets in the network	40
Number of services provided by the network	5
Value of ε_1_, ε_2_, ε_3_	0.5, 1, 0.5
Initial energy of each node	500 units
Energy cost for collecting a service data during an epoch	1 units
Fraction of survived nodes	75%
Value of *sgp*	0.6

**Table 3.** The impact of the network size on the noise level.

**Network Size**	**100**	**200**	**300**	**400**	**500**
Noise-corrupted Data	90.0	176.1	252.9	327.2	416.8
Denoised Data	44.8	48.4	49.1	50.1	49.6
